# Health care professionals’ experience, understanding and perception of need of advanced cancer patients with cachexia and their families: The benefits of a dedicated clinic

**DOI:** 10.1186/s12904-016-0171-y

**Published:** 2016-12-30

**Authors:** David Scott, Joanne Reid, Peter Hudson, Peter Martin, Sam Porter

**Affiliations:** 1 School of Nursing and Midwifery, Queen’s University Belfast, 97 Lisburn Road, Belfast, BT9 7BL UK; 2 Barwon Health, Geelong, VIC Australia

## Abstract

**Background:**

Cachexia is defined as the on-going loss of skeletal muscle mass that cannot be fully reversed by conventional nutritional support. It is found in up to 80% of patients with advanced cancer and has profound psycho-social consequences for patients and their families. Previous studies demonstrate that many healthcare professionals receive little formal education in cachexia management leading them to feel that they have limited understanding of the syndrome and cannot intervene effectively. This study aims to examine the value of a dedicated cachexia clinic and its influence on staff understanding and practice.

**Methods:**

An exploratory qualitative study was conducted. The study employed semi-structured interviews with a range of healthcare professionals responsible for designing and delivering cancer care in a large teaching hospital in Australia. This hospital had a dedicated cachexia clinic.

**Results:**

In-depth interviews were conducted with 8 healthcare professionals and senior managers. Four themes were identified: formal and informal education; knowledge and understanding; truth telling in cachexia and palliative care; and, a multi-disciplinary approach. Findings show that improved knowledge and understanding across a staff body can lead to enhanced staff confidence and a willingness to address cancer cachexia and its consequences with patients and their families.

**Conclusion:**

Comparisons with similar previous research demonstrate the advantages of providing a structure for staff to gain knowledge about cachexia and how this can contribute to feelings of improved understanding and confidence necessary to respond to the challenge of cachexia.

**Electronic supplementary material:**

The online version of this article (doi:10.1186/s12904-016-0171-y) contains supplementary material, which is available to authorized users.

## Background

Cachexia is a multi-dimensional syndrome found in up to 80% of patients with advanced cancer. It has been consensually defined as ‘an ongoing loss of skeletal muscle mass (with or without loss of fat mass) that cannot be fully reversed by conventional nutritional support’ [1–4]. Cancer cachexia can develop through three stages: pre-cachexia, cachexia and refractory cachexia. Refractory cachexia refers to the ‘stage where reversal of weight loss seems no longer possible’ [2]. At present, although there are an increasing number of studies related to cancer cachexia, its underlying mechanisms are still poorly defined, and therapeutic options remain limited [5].

Previous studies highlight that cachexia in advanced cancer has profound bio-psycho-social consequences for patients and their families [6–9]. Physically, loss of muscle mass often leads to extreme weakness and decreased functional ability [4, 10]. Psycho-socially, cachexia can have negative consequences for the patient’s body image, contributing to social isolation and emotional distancing [11]. In addition, the accompanying symptom of anorexia often creates tension between patients and their family members, who focus on feeding to reverse the patients’ decline [8, 12].

The experiences of patients and family carers have been explored in a number of qualitative studies [7, 8, 11, 13, 14] however, few studies have explored the views of health care professionals regarding cachexia management [15, 16]. Existing research highlights that cancer cachexia has been relatively under-researched and under-resourced [4] and its association with advanced and incurable disease, coupled with a lack of potential clinical treatment has led to a reluctance amongst healthcare professionals to discuss the issue with patients and/or their carers [4, 14–16].

### Aim

The aim of this study was to examine the views and experiences of healthcare professionals who worked in an institution that had a dedicated cachexia clinic in Melbourne, Australia and, establish how undertaking work placements in this multi-disciplinary clinic impacted on their understanding and management of cachexia. In addition we set out to compare and contrast the results obtained in Australia with previous studies undertaken in the UK [15–18]. The clinic had been developed to work on a bi-weekly basis and had core staff which included a Palliative Care Physician, a Nurse, a Physiotherapist and a Dietician. Referrals were received from GPs, hospital based physicians, nursing staff and other allied health professionals such as physiotherapists and dieticians. The clinic provided an initial 40 min assessment which included anthropometry, nutritional self-assessment (using the Patient—Generated Subjective Goal Assessment tool PG-SGA), upper and lower limb strength assessments and relevant pathology. Based on the best available evidence, the team would develop a multi-modal treatment regime which included nutritional, exercise and pharmacological treatments tailored to the individual requirements of each patient. Patients were then reviewed on a regular basis to establish how cachexia was developing and to amend treatment.

## Method

Participants comprised senior healthcare professionals and managers responsible for cancer care in a teaching hospital in a major Australian city (*n* = 8). Potential participants were approached via a professional colleague with a dual academic appointment and given an invitation letter, information sheet and consent form. Written informed consent was obtained prior to interview; pseudonyms are employed to ensure confidentiality.

Participants were identified using purposive sampling ensuring they reflected the full range of professionals involved in the planning and delivery of services for cachexia care.

Data collection comprised semi-structured interviews undertaken by two members of the research team (S.P. and J.R.) in November 2014. The interview schedule was informed by previous research undertaken by members of the research team [15, 16, 18] and focused on 4 main areas: understanding cachexia; managing cachexia; multi-professional working; and, the needs of patients and families (see Additional file 1). All interviews employed a reflective questioning style to ensure they accurately represented the thoughts and opinions of participants. All interviews were conducted in private offices and were digitally recorded before being transcribed for analysis. Interviews lasted between 24 and 50 min.

### Analysis

Data were subjected to a thematic analysis as this method is suited to the exploration of relatively unknown topics [19]. This study did not set out to examine a predetermined hypothesis or to test a theory; it sought to generate qualitative data that would provide a better understanding of what was a novel service. This study will assist the development of an ongoing programme of research which aims to develop materials to assist the future training of healthcare professionals. As such, we were not concerned with issues such as data saturation. Analysis was aided by the use of Nvivo 15 computer software and followed the procedures for thematic analysis described by Hayes [20] and Braun and Clarke [21], see Table 1. All authors contributed to the analysis and final themes were selected to accurately reflect the views and experiences of participants.

**Table 1:** Phases of thematic analysis

Phase		Description of process
1	Familiarise self with data	Transcribe data, read and re-read noting and discussing initial ideas
2	Generate initial codes	Code interesting features of data in systematic way and collate data relevant to each code.
3	Search for themes	Collate codes into potential themes, gather all data relevant to each theme.
4	Review themes	Check to ensure codes reflect content of dataset, generate thematic maps of dataset
5	Define and name themes	Refine specific content of each theme, generate clear definitions and name themes
6	Produce report	Select extract examples reflecting the content of the analysis and which answer the research question.

## Results

Results across the eight interviews were broadly consistent and overall, four themes emerged: Formal and informal education; knowledge and understanding; truth telling in cachexia and palliative care; and, a multi-disciplinary approach.

### The contribution of formal and informal education

While respondents reported that formal training in cachexia had not been a component of their clinical training, all were working in a hospital that had a dedicated cachexia clinic and most (*n* = 6) had benefitted from previously working in this clinic as part of their in-house training. The clinic had been established by a senior clinician in their hospital. Consequently, many staff throughout the hospital had gained exposure to cachexia assessment and management strategies. This practice appeared to have led to improved understanding and provided staff with the knowledge and confidence that was required to identify and discuss cachexia, and its implications, with patients and their families.

*“So every Resident, Registrar and Nurse, whoever goes through [hospital] probably gets drilled in the importance of cancer cachexia. So, he’s [lead clinician] got a particular set-up there where he’s got an outpatient clinic servicing patients with cachexia in the region where there is a medical or nursing person, a dedicated dietician, and a dedicated physiotherapist who, every week they have a clinic on a Tuesday afternoon where they see new and review patients and so, it’s a great service”* (Doctor S1).

Having gained an understanding of the significance of cachexia through placements in the clinic, respondents reported that they were more mindful of identifying the syndrome and the knowledge gained during these work placements influenced their future professional practice.

### Knowledge and understanding

Respondents demonstrated detailed knowledge and awareness of cachexia recognising it as a distinct clinical syndrome which presented unique challenges to both the patient and the healthcare professional.

*“It’s the loss of skeletal muscle…and it is associated with inflammatory mediators and obviously associated with cancer whatever the underlying condition may be…”.* (Doctor J1)

*“It’s very common with advanced malignancy, decreased appetite is common in people as a presenting complaint in malignancy but the more advanced the disease becomes then, I think you get up to 80% of advanced malignancy complain of or suffer from cachexia”.* (Doctor A1).

### Truth telling in cachexia and palliative care

Healthcare staff reported that they sought to identify cachexia and initiate discussions with both the patient and carers at an early time point as they recognised the psycho-social importance of addressing cachexia. Respondents also indicated that they employed a consistent team-based approach when discussing cachexia with patients and their families.

*“We don’t ignore it or say ‘sorry, that’s bad luck’. We do have access to two dieticians, in our two inpatient units, so we do take steps, we do get more assessed physio and OT; we talk about it in our team meetings. It’s like doing a needs assessment, we look at what the person per se sees as important”.* (Doctor J2)

*“I guess the first thing would be education, in terms of my management of the situation. It would be explaining to the patient and their family what is happening, that this is associated with the cancer and not necessarily how much they are eating…it’s a complex metabolic process”.* (Doctor J1)

Respondents reported being open and honest in discussing cachexia although they recognised the sensitivity of the diagnosis as they sometimes employed strategies which allowed the patient to control the amount of information they received.

*“Over the years you learn ways around these things* [discussing prognosis] *you know? We’re not going to tell such and such that, if they don’t want to know it, but we need to ask him does he want to know it? And, if they tell us they don’t want to know, speak to him, then, for him that’s fine, that’s not a problem, but we need to sort of answer that question” (Nurse, S4).*

Respondents discussed the type of interventions they typically employed with patients. Most commonly this comprised the provision of information to patients and carers in order to help them focus on accepting their diagnosis and ensuring the patient did not feel under pressure to eat.

*“It’s education that is the key and that’s where I normally start rather than reaching for a script pad and pen. It’s often about just sitting people down and talking, or, in fact listening, hearing what it is like for them, what it feels like to deal with these problems”.* (Doctor S1)

### A multi-disciplinary approach

Respondents demonstrated a clear understanding and awareness of cachexia across a range of healthcare professions and, as such, they had been able to employ a multi-disciplinary approach which integrated cachexia management into routine care. Respondents did recognise that this approach was not typical of other Australian hospitals but was due to the influence of a senior clinician who championed cachexia locally and had developed a specialist cachexia clinic. Previous placements in this specialist setting had facilitated the dissemination of knowledge through a wide range of healthcare staff.

*“So now it’s a case that, when we go and see patients, it’s in our head, it’s something we think about…can we give them this medication? Can we manage it pharmacologically? Or, can we do other things that will help?”* (Nurse S4)

*“I would do it, the nurses would do it, the registrars would do it. So we would reinforce, we would definitely all be aware of it and try to settle the situation a bit. Take the focus off the food and it’s always something I talk about when someone has a poor appetite, you know? Try to remind them that there comes a stage in someone’s illness where eating should be about pleasure and quality of life and they shouldn’t feel that it should be a burden to them”.* (Doctor J1)*.*

Respondents recognised the limitations of this localised approach and believed there was a need for more structured learning opportunities for healthcare staff to ensure that they acquired the specialist knowledge required to intervene effectively with this client group.

## Discussion

Comparing results obtained in this study with similar research helps clarify how educational opportunities, culture and organisational context can have a significant effect on the care of advanced cancer patients.

This research demonstrates that, despite not having a significant level of formal education about the care of patients with cachexia the consequences of this knowledge gap were mitigated by the experiential knowledge that was systematically gained through placements in a specialist clinic. Although there has been relatively few studies in this area, previous research indicates that many professionals find managing cachexia in patients with advanced cancer challenging [17, 22, 23]. Qualitative research carried out in the UK found that healthcare professionals’ professional practice was influenced by a combination of their existing knowledge, the culture within their clinical area and the availability of resources; variation in these areas contributed to inconsistent and potentially sub-optimal care [14, 15, 17]. In addition, studies found distinct differences in the manner in which palliative care specialists and non-palliative professionals responded to the challenge of cachexia with many non-specialist professionals reporting that they had insufficient understanding to intervene effectively [15, 18, 24]. Previous research has indicated that, in the absence of effective clinical treatments, healthcare professionals were reluctant to discuss cancer cachexia and it’s prognosis with patients and/or their families [16, 17, 24]. Findings from the current study highlight a number of differences in the manner in with cancer cachexia was identified, prioritised and managed in this Australian hospital when compared to other research [2, 14, 17, 25]. In particular, evidence from Melbourne suggests that professionals had sufficient understanding to recognise the signs and symptoms associated with cachexia and had been able to refer patients to a specialist clinic. This dedicated clinic had centralised expertise and, through a policy of providing clinical placements had been able to disseminate knowledge throughout the workforce and influence the clinical ‘culture’ within the hospital. The creation of this clinic appeared to have influenced the manner in which healthcare professionals responded to patients with cachexia..

Existing studies illustrate that healthcare professionals receive limited formal education about cachexia at both undergraduate and postgraduate level and have limited understanding of the syndrome [15, 17, 23]. This lack of formal education and understanding coupled with the lack of a standardised assessment tool contributes to an inability to consistently address the needs of patients with cachexia [15, 17]. Previous UK based studies demonstrated that professionals gained knowledge about cachexia through experience on general, oncology, haematology and palliative care wards [15, 16, 18]. These diverse environments contribute to inconsistent learning opportunities both within and between various professional groups [15, 26]. Participants in Melbourne reported that they also had limited formal education in cachexia. However, the specialist cachexia clinic had provided an environment where expertise was centrally located providing an important service to patients and their families and also provided a consistent learning environment which had facilitated greater understanding and had contributed to a more consistent approach. Globally, it is acknowledged that in order to ensure best possible care there is a need to recognise cachexia as a specific syndrome [1] requiring specific care and our results suggest that it may be beneficial to incorporate cachexia education into teaching at both pre-registration and post-registration levels [27, 28].

Similar to previous international research, participants in this study recognised that patients and their families required information to help them cope with the bio-psychosocial stressors associated with cancer cachexia [7, 13, 29, 30]. Previous research indicated that many healthcare professionals find communicating with patients and their families about cachexia difficult [15, 17, 24] and could lead to uncomfortable discussions about end-of-life issues which they often felt ill-prepared and insufficiently resourced to undertake [16, 18]. This contrasts with the Australian experience where staff reported being competent and at ease when discussing the totality of patient needs including end-of-life issues. This study adds to a growing body of literature highlighting a need to educate healthcare professionals on how to initiate conversations about end-of-life issues in order to provide holistic patient centred care throughout the treatment journey [15, 24, 31]. The implementation of techniques such as motivational interviewing, solution-focused brief therapy and cognitive behavioural therapy may help healthcare professionals achieve this goal [15, 24]. In addition, the need to provide co-ordinated patient-centred care is illustrated by the Australian experience where respondents reported a greater awareness of the positive impact of supportive care measures [30] and felt this contributed to a more inclusive, multi-disciplinary approach to communicating with patients and carers. Following best available evidence, staff in Australia further recognised the importance of providing information to patients and their families in order to de-emphasize the role of food and to ensure that meal times and food consumption did not become a source of conflict between patients and their families [13, 26, 32].

Cachexia in advanced cancer is known to have profound psycho-social implications for patients and family members [3, 7, 33]. Best evidence suggests that an appropriate response requires a holistic, patient-centred approach to meet the multi-dimensional needs of this patient group [23, 24, 34, 35]. The approach which had been employed in this Australian hospital indicates that general and specialist nurses, doctors, dieticians and physiotherapists all have a role to play in ensuring a holistic patient-centred experience.

## Conclusions

This study highlights the importance of recognising cachexia as a significant syndrome in palliative care and of providing a specific service responses to address the needs of patients and/or carers. It further demonstrates that knowledge and understanding of cachexia and its effects on patients is essential for the provision of high-quality care.

Comparing results from this study with the existing research demonstrates that without this knowledge and the confidence that it generates, many healthcare professionals found it extremely difficult to discuss cachexia with patients and their families. This study and previous research illustrates the need to develop specialist communication skills training for healthcare professionals to ensure they develop the skills necessary to sensitively deliver this information. It further emphasises that the care of patients with cachexia and their families should not be the responsibility of a single profession, but should employ a multi-disciplinary approach where a knowledgeable staff body can provide a clear and consistent message.

In common with other healthcare systems, participants from Australia had received little formal educational in cancer cachexia. However, the provision of work placements in a dedicated clinic, had facilitated a learning environment that allowed best practice to develop across and between professional groups. While the spread of dedicated clinics would certainly be a welcome development, it is uncertain whether such an intensive model is amenable to large-scale adoption. While providing an exemplar for clinical approaches to the care of those with cachexia, the success of the Australian model described here does not negate the need for formal educational preparation of clinicians to ensure that they are capable of engaging effectively with patients and their families to support them in coping with this distressing syndrome.

## Additional file

Additional file 1: Interview guide. (DOC 140 kb)
