# Supplementary material for: Health care professionals’ experience, understanding and perception of need of advanced cancer patients with cachexia and their families: The benefits of a dedicated clinic
Source: BMC Palliat Care. 2016 Dec 30;15:100. doi: 10.1186/s12904-016-0171-y (PMC5203721; doi:10.1186/s12904-016-0171-y)
Supplement: Additional file 1: — Interview guide. (DOC 140 kb) [file 12904_2016_171_MOESM1_ESM.doc]

|  | | |
| --- | --- | --- |
|  | |  |
|  |  |  |
|  |  |  |
|  |  |  |
|  |  |  |
|  |  |  |
|  |  |  |

Additional file 1

Semi-structured interview agenda

Aims:

Health care professionals’ perceptions of cancer cachexia;

How they care for patients with advanced cancer who have cachexia;

What they perceive the needs of cachectic patients and their families to be

1) Introductions and consent

2) Interpreting cachexia

Can you tell me what you think of when you hear the term ‘cachexia’?

What images come to mind when you think of someone as having cachexia in advanced cancer?

Can you explain why these changes occur?

3) Managing cachexia

Can you tell me about your experience of managing an advanced cancer patient with cachexia?

Are you aware of any interventions in place for patients with cachexia in advanced cancer?

How would you assess a patient with advanced cancer for cachexia?

What are your priorities of care for an advanced cancer patient with cachexia and their families? Why these priorities?

4) Multi-professional working

What support do you have in managing patients with cachexia in advanced cancer and their families?

What do you consider is the most important part of your role in managing patients with cachexia and their families? Why?

5) Needs of patients and their families

Do you consider cachexia in advanced cancer to be problematic for patients and their families? If so, why? If not, why?

What do you perceive is the impact of cachexia in advanced cancer on patients and their families? Why is this?

What do you think the needs of advanced cancer patients with cachexia and their families are? Why do you think this?

6) Quality management of cachexia

How do you perceive the current care available for advanced cancer patients with cachexia?

What would you consider to be good quality cachexia management?

Can you think of anything else we should be doing?

Anything else you would like to add to the discussion which we have not covered?
